# Full Inactivation of Human Influenza Virus by High Hydrostatic Pressure Preserves Virus Structure and Membrane Fusion While Conferring Protection to Mice against Infection

**DOI:** 10.1371/journal.pone.0080785

**Published:** 2013-11-25

**Authors:** Carlos H. Dumard, Shana P. C. Barroso, Guilherme A. P. de Oliveira, Carlos A. M. Carvalho, Andre M. O. Gomes, José Nelson S. S. Couceiro, Davis F. Ferreira, Dirlei Nico, Andrea C. Oliveira, Jerson L. Silva, Patrícia S. Santos

**Affiliations:** 1 Instituto de Bioquímica Médica and Instituto Nacional de Ciência e Tecnologia de Biologia Estrutural e Bioimagem, Universidade Federal do Rio de Janeiro, Rio de Janeiro, Rio de Janeiro, Brazil; 2 Instituto de Microbiologia Paulo de Góes, Universidade Federal do Rio de Janeiro, Rio de Janeiro, Rio de Janeiro, Brazil; Center for Biologics Evaluation and Research,, United States of America

## Abstract

Whole inactivated vaccines (WIVs) possess greater immunogenicity than split or subunit vaccines, and recent studies have demonstrated that WIVs with preserved fusogenic activity are more protective than non-fusogenic WIVs. In this work, we describe the inactivation of human influenza virus X-31 by high hydrostatic pressure (HHP) and analyze the effects on the structure by spectroscopic measurements, light scattering, and electron microscopy. We also investigated the effects of HHP on the glycoprotein activity and fusogenic activity of the viral particles. The electron microscopy data showed pore formation on the viral envelope, but the general morphology was preserved, and small variations were seen in the particle structure. The activity of hemagglutinin (HA) during the process of binding and fusion was affected in a time-dependent manner, but neuraminidase (NA) activity was not affected. Infectious activity ceased after 3 hours of pressurization, and mice were protected from infection after being vaccinated. Our results revealed full viral inactivation with overall preservation of viral structure and maintenance of fusogenic activity, thereby conferring protection against infection. A strong response consisting of serum immunoglobulin IgG1, IgG2a, and serum and mucosal IgA was also detected after vaccination. Thus, our data strongly suggest that applying hydrostatic pressure may be an effective method for developing new vaccines against influenza A as well as other viruses.

## Introduction

Seasonal influenza virus infections cause significant morbidity and mortality worldwide [1,2]. In addition, pandemic influenza strikes periodically, infecting a large number of people and potentially causing many deaths [3]. Since 1977, the H1N1 and H3N2 viruses have co-circulated globally and are responsible for seasonal epidemics that have caused an average of 36,000 deaths annually in the U.S. alone [4]. Prevention is considered to be the most effective method of reducing the socio-economic burden of influenza [1,3]. The currently available human vaccines are primarily trivalent subunit vaccines, containing 2 influenza A and 1 influenza B subtype [5]. Whole virus vaccine formulations have been shown to be more immunogenic in a naive population and may be needed in a pandemic situation to elicit an adequate immune response [6]. Furthermore, many studies have demonstrated that whole inactivated influenza viruses are more immunogenic than split or subunit vaccines [7-9].

Globally, there is a need for new vaccine types that are more effective, non-invasive, safe, and ideally that can be produced faster and at a low cost. Pressure-based virus inactivation is a promising alternative and an industrially mature technology. Some groups have successfully inactivated human and animal viruses using high hydrostatic pressure (HHP), and satisfactory immune responses have been produced after vaccination and challenge [10-12]. 

HHP is a non-thermal, energy-efficient technology that has been applied to viruses for the purpose of stability studies and viral inactivation [13-15]. HHP has been a useful tool in studies that disturb viral macromolecular structures, which has led to an improved understanding of viral particles [16,17]. HHP is unique in its ability to change the volume of the molecules, thereby disturbing these structures and leading to dissociation and denaturation processes [18].

Viruses that have been successfully inactivated by HHP include vesicular stomatitis virus [11], yellow fever virus [15], avian influenza viruses [10,19], Hepatitis A virus [20], norovirus [21], and infectious bursal disease virus [12]. HHP has the potential to cause viral inactivation without drastically affecting viral immunogenic properties or destroying structural epitopes [10,11,19,22]. This interesting finding highlights the potential application of this tool to prepare whole viral vaccines in a simple, fast, and inexpensive way. Moreover, this approach would not introduce exogenous substances into vaccines, which differs from inactivation using chemical methods [15,12,11].

Here, we describe the effects of HHP on the structure and the biological and functional activities of the influenza X-31 virus. For structural analyses, fluorescence spectroscopy, light scattering, and electron microscopy were used. For functional analyses, the viruses were assayed for their hemagglutinin (HA), neuraminidase (NA), and membrane fusion activities. To verify viral inactivation by HHP, we measured the virus titer in cells and performed an RT-PCR assay.

We found that HHP was able to fully inactivate the influenza virus while preserving its overall structure and fusogenic activity, and this method of inactivation also protected vaccinated mice against infection. Our data strongly support the application of HHP to the development of new vaccines for influenza A as well as other viruses.

## Materials and Methods

### Ethics statement

 All experimental procedures were approved by the Institutional Animal Ethics Committees under the Federal University of Rio de Janeiro welfare assurance number IBqM065. All procedures were performed under isoflurane anesthesia, and all efforts were made to minimize suffering.

### Virus

 Virus stocks of influenza virus X-31 (H3N2) (a reassortant strain of A/Aichi/2/68 and A/PR/8/34) were prepared by infecting 10-day-old specific pathogen-free (SPF) chick embryo eggs with 0.1 ml of virus at a 100-fold dilution of a 128 HA unit stock. After 48 h of incubation, allantoic fluid was cleared by low-speed centrifugation (6,000 x *g*) for 30 min. The pellet was discarded, and the supernatant was concentrated by centrifugation at 100,000 x *g* for 1 h. The pellet was resuspended in PBS pH 7.4 at a ratio of 1 ml of PBS to 100 ml of initial allantoic fluid. To purify virus, we carried out sucrose gradient purification. Initially, sucrose was diluted in PBS pH 7.4 to different concentrations, and a sucrose gradient with bands (2 ml per band) varying from 20% to 60% density (with an interval of 10% density) was constructed. Concentrated samples were placed on the 20% sucrose fraction and centrifuged for 2 h at 100,000 x *g*. After centrifugation, the fraction between 50% and 60% was collected and stored at -80°C. All centrifugation steps were carried out at 4°C.

### Hemagglutination assay

 The virus preparations were assayed for their hemagglutinating (receptor-binding) activity in 96-well micro-titer plates (Nunc, Roskilde, Denmark - U type). Twenty-five microliters of PBS were added to each well, then 25 µl of the viral suspension was added to the first well in each column, and serial dilutions were made by transferring 25 µl from the first well of each column to the successive wells. The final 25 µl was discarded. The positive control was lectin, and the negative control was PBS. Finally, 25 µl of 0.5% chicken erythrocyte suspension was added to each well on the plate, and the hemagglutinating titers were recorded after 45 min as described previously [23].

### Measurement of neuraminidase activity

 The NA assay was employed to test the effect of HHP on virus NA activity. Virus solutions of 5 µl were mixed and incubated with MES buffer at 37°C for 30 min. Next, 15 µl of substrate solution (4-MU-NANA; 2´-(4-methylumbelliferyl)-A-D-N-acetylneuraminic acid sodium, Sigma) was added, and the mixture was incubated at 37°C for 1 h, protected from light. Fluorescence was then measured (excitation 365 nm, emission 460 nm), and relative activities were calculated as described by Song (2005) [24].

### Cell culture and virus infection

 Madin-Darby Canine Kidney (MDCK) cells obtained from the Rio de Janeiro Cell Bank were cultured in Dulbecco's modified Eagle medium (DMEM; Invitrogen, Carlsbad, CA, USA) containing low glucose and supplemented with 10% fetal bovine serum (Invitrogen, Carlsbad, CA, USA). Before infection, 80–90% confluent cells were washed with PBS to remove the FBS, infected with 100 µg of virus diluted in serum-free DMEM containing 2 µg of trypsin, and incubated for 1 h at 37°C. After this period, the infection medium was removed, the cells were washed with PBS, and new culture medium containing 2 µg of trypsin was added. The period of infection was 48 h.

### Tissue Culture Infective Dose (TCID_50_)

The infectivity of the influenza virus was measured according to the 50% TCID_50_/ml in MDCK cells. Cells were infected with serial dilutions ranging from 10^-1^ to 10^-8^, with the infection medium containing 2 µg of trypsin. After 48 h at 37°C, the cytopathic effects of the influenza virus were observed under the microscope, and the TCID_50_/ml was calculated according to the Reed and Muench method [25].

### Serial passages

The residual infectivity of the pressurized virus samples was assayed for 3 sequential serial passages. For each blind passage, the samples, which revealed the absence of infectivity measured by TCID_50_/ml, were inoculated into an MDCK cell monolayer. The total cellular RNA was analyzed by RT-PCR assay. Infection proceeded with the second culture being infected with 100 µl of the supernatant from the first culture and the third culture being infected with 100 µl from the second culture. At all serial passages, the cell medium contained 2 µg of trypsin.

### RT-PCR assay

MDCK cells were grown to approximately 80–90% confluence and then infected with X-31 at a protein concentration of 100 µg/ml. After 48 h of infection, the supernatants were removed and cells washed with PBS. Cells were scraped off and collected by low-speed centrifugation. RNA isolation was performed using the Trizol reagent (Invitrogen, Carlsbad, CA, USA). First-strand cDNA (2 µg) synthesis was performed as previously described [26]. PCR reactions were carried out using 200 nM primers, 1.5 mM deoxynucleotide triphosphates (dNTPs), and 2 U of Taq DNA polymerase (Phoneutria, Belo Horizonte, MG, Brazil). The PCR cycling conditions were based on those reported by Daum [27]. The primers used in this paper were also based on those reported by Daum and were developed to a conserved region of the HA1 portion and optimized to the viral subtype used in this paper: F-AACGGAACACTAGTAGTGAA 3’; R-TCAACCAGTTCAGTCTAC 3’. PCR products were visualized in 1% agarose gels stained with ethidium bromide. 

### HHP apparatus and procedure

 The HHP vessel contains a cylindrical body and is made of Vascomax 300. Samples were placed in the interior of the pressure vessel (volume of 10 ml) in a fused quartz cylindrical cell with an approximate volume of 1.5 ml and diameter of 10 mm. The pressure vessel contains windows necessary for spectroscopic and light scattering measurements, which are made of fused quartz with the following dimensions: diameter, 0.5 in; thickness, 0.30 in. The cell is sealed with a polyethylene stopper that permits equalization between the hydrostatic medium and the sample inside the cell. The pump used (High Pressure Equipment Company/model 37.- 5.75-60 – Erie, PA, USA) is a manual pressure generator and was designed for applications where a liquid is to be compressed within a small volume to create pressure. The pressure gauge (ASCROFT – Stratford, ON*, Canada*) contains a scale in psi with a maximum rate of 50.000 psi and a minimum graduation of 50 psi. The time taken to reach 289.6 MPa from ambient pressure was 3 min on average. The experiment time began when the desired pressure was reached. Decompression took the same average time. The pressure vessel was coupled to a thermostatic bath used to keep the temperature at 25°C. The thermostatic fluid was ethylene glycol. To monitor the cell temperature, a conduit for a thermometer was drilled into the pump. For more details about the HHP equipment, consult the report of Paladini and Weber [18]. Ethanol was used as the pressure-transmitting fluid. During pressurization, care was taken to prevent the formation of air bubbles.

### Spectroscopic measurements

Fluorescence spectra and light scattering were recorded using an ISS PC spectrofluorimeter (ISS Inc, Champaign, IL, USA). The intrinsic fluorescence of aromatic residues was obtained by excitation at 280 nm, and emission was observed from 300 to 420 nm. Scattered light (320 nm) was detected at a 90° angle of the incident light by integrating the intensity in the 315-325 nm window. This wavelength was chosen because proteins and nucleic acids do not absorb at 320 nm. Fluorescence spectra at pressure *p* were quantified by spectral mass center <V_*p*_ *>*:

$$<{\text{V}}_{p}>=\sum V_{i}F_{i}/\sum F_{i}^{}$$

where Fi is the fluorescence emitted at wavelength *vi*. Structural perturbations were also evaluated according to the fluorescent probe bis-8-anilino-1-naphthalenesulfonate (bis-ANS) (Molecular Probes – Eugene, OR, USA) (15 mM) fluorescence. Samples were excited at 360 nm, and emission was collected in the range of 400–600 nm.

### Electron microscopy (EM)

The visualization of pressurized and control viruses was performed in a Morgani transmission electron microscope operated at 100 kv. Copper grids, coated with carbon and containing 300 µg/ml of sample, were treated with a contrasting solution of uranyl acetate at 2%. After acquisition, the images were similarly processed for brightness/contrast with Adobe Photoshop for better viewing.

### Fusion assay

Fusion between influenza virus and the endosomal membranes of target cells was evaluated by laser-scanning confocal fluorescence microscopy using virus particles labeled with 1,1’- Dioctadecyl-3,3,3’,3’-Tetramethylindodicarbocyanine Perchlorate (DiD) (Invitrogen, Carlsbad, CA, USA). DiD is a fluorescent membrane probe that presents a self-quenching behavior and therefore does not fluoresce in saturating conditions. Thus, the occurrence of membrane fusion can be inferred by means of DiD fluorescence dequenching. Fluorescent labeling of influenza virus particles was performed by incubating 100 µg of purified virus with 24 µM DiD in a total volume of 100 µl of low-glucose DMEM for 10 min at room temperature. Unincorporated dye molecules were removed by centrifugation in a 100-kDa cutoff Amicon Ultra filter unit (Millipore, Billerica, MA, USA). Labeled virus particles were suspended in PBS and passed through a 0.22-µm filter to remove viral aggregates. MDCK cells cultured in an 8-well chamber slide system (Nunc, Roskilde, Denmark) were washed with PBS and infected with DiD-labeled influenza virus for 30 min at 37°C in a 5% CO_2_ atmosphere. Cells were subsequently washed again with PBS, fixed with 3.7% formaldehyde, and then visualized on an LSM 510 META inverted microscope (Zeiss, Jena, Germany) with excitation by a helium-neon laser at 633 nm and emission collected from 650-710 nm using a plan-neofluar 40x/1.30 oil immersion objective.

Images were acquired by an assessor who was blinded to the identity of the samples. Ten fields were acquired for each experimental condition, and the representative fields are shown. Fusogenic activity relative to control images was obtained by considering the fluorescence intensity of cells infected with control virus (native virus) as 100% fusion efficiency.

### Mice

Adult (6-week-old) female BALB/c mice were obtained from Laboratory Animals Breeding Center (Cecal)/Fundação Oswaldo Cruz (FIOCRUZ), and housed in groups of 5 animals with free access to food and water. Prior to inclusion in experiments, mice were acclimatized for 7 days.

### Inactivation, immunization and challenge

 Inactivation by HHP was carried out by viral sample pressurization for 3 h at 289.6 MPa at 25°C in phosphate saline buffer (PBS) at pH 7.4. Prior to immunization and challenge, mice were anesthetized by isoflurane inhalation. Mice were immunized via intranasal (i.n.) or intramuscular (i.m.) route with 40 µl of concentrated and filtered (0.22 µm) virus diluted in PBS (20 µg of total protein concentration per dose). Control groups received 40 µl of PBS i.n or i.m route. An interval of 14 days was included between the first and the second doses. Fourteen days after the second dose, mice were challenged intranasally with 40 µl of concentrated and filtered (0.22 µm) virus diluted in PBS at a 4,096 HA titer. After challenge, the mice were monitored daily for weight loss for 12 days. Weight loss monitoring was carried out with 5 animals per group.

Euthanasia

 Mice were first anesthetized by isoflurane inhalation followed by CO_2_ inhalation.

### Detection of influenza-specific antibodies

Influenza-specific serum immunoglobulins (Igs) and IgA obtained from nasal washes were detected by ELISA. Samples from individual animals (n=8 per group) were assayed for IgG2a, IgG1, and IgA. The measurements were made 14 days after the first and second doses. The ELISA protocol has been previously described [28]. Briefly, 96-well ELISA plates (Greiner, Germany) were coated with 50 µl (2 µg/ml) of X-31 virus (H3N2) overnight at 4°C (50 µl/well). Influenza-specific antibodies were detected using anti-mouse IgA, IgG1, and IgG2a antibodies conjugated to peroxidase (Southern Biotechnology Associates, Birmingham, *AL, USA*) at a dilution of 1:1,000. The reaction was developed with O-phenylenediamine (Sigma, St Louis, MO, USA), interrupted with 1 N sulfuric acid, and monitored at 492 nm. Each individual serum sample was analyzed in triplicate in double-blind tests. Positive and negative control sera were included in each test. Results were expressed as the mean of the absorbance values (492 nm).

### Hemagglutination Inhibition (HI) Assay

Neutralizing antibodies were also measured in the sera using an HI assay, which was carried out as described in the WHO Manual on Animal Influenza Diagnosis and Surveillance [29]. Briefly, serum samples (n=8 per group) were serially diluted in PBS, then mixed with aliquots of virus corresponding to 8 HA units in U-bottom 96-well plates (Nunc Roskilde, Denmark), and incubated for 60 min at room temperature. At the end of the incubation, 1.0% turkey red blood cells were added and incubated for a minimum of 30 min. The serum HI antibody titer of a given sample was defined as the reciprocal of the last serum dilution that completely inhibited hemagglutination.

### Statistical Analysis

Results were expressed as the mean value and corresponding standard deviation of individual results.

The normal distribution of values of each variable was assessed by the Anderson Darling A2 test (Analyze-it). Statistical analysis was performed using the software SPSS (for windows). Comparisons between and within groups were analyzed by analysis of variance test (one-way ANOVA) and Tukey’s multiple comparison test.

## Results

### HHP completely eliminates viral infectivity

**Figure 1:**
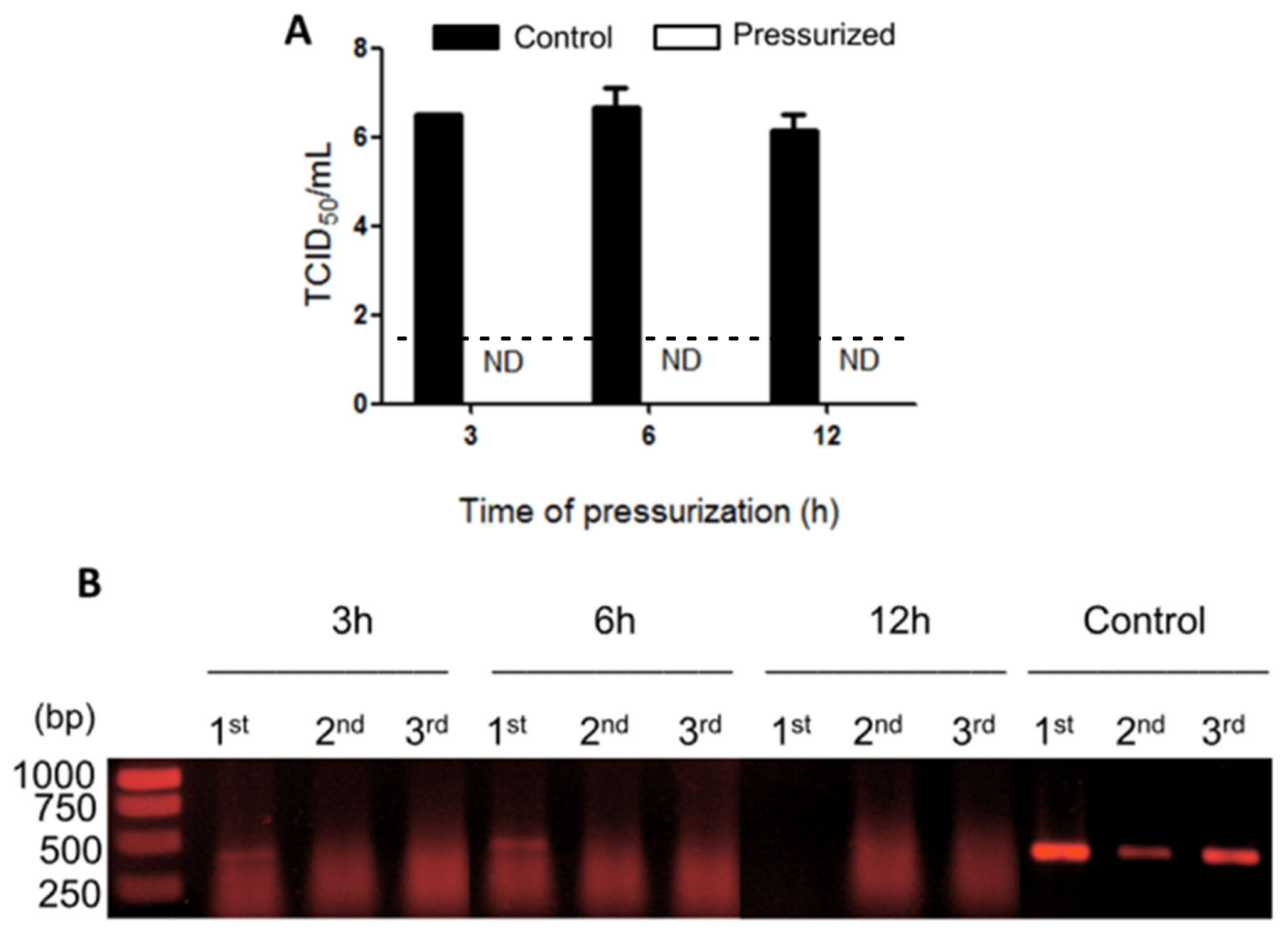
Influenza virus is inactivated by HHP. In this assay, the virus sample was used at a final concentration of 100 μg/ml. The virus was pressurized over different intervals at 289.6 MPa and 25°C. (A) MDCK cell monolayers were infected with virus dilutions ranging from 10^-1^ to 10^-8^. TCID_50_ values was calculated using the Reed-Muench method. ND = not detected. (B) RT-PCR results for the serial passages of pressurized virus. 1^st^, 2^nd^, and 3^rd^ refer to the sequence of passage. Control = non-pressurized virus kept at 25°C for 12 h. The dashed line indicates the cut-off value for obtaining a positive result.

Figure 1A shows the effect of pressure on influenza virus infectivity, as determined by the TCID_50_/ml assay in MDCK cells. No cytopathic effect was observed even after 3 serial passages in the MDCK cell culture. These data revealed that pressure treatment left the virus fully inactivated.

To ensure that the virus particles were inactivated after the pressure treatment and TCID_50_/ml assay, we performed RT-PCR (Figure 1B) using a primer that amplifies a conserved region of the HA1 portion. Total RNA was extracted from cells, avoiding the RNA in the supernatant, and the viral RNA was amplified. This was done to avoid the amplification of inactivated virus that may have remained in the supernatant due to the serial passages. After 3 h of pressurization, the virus had completely lost its infection ability. For the first serial passage, a discrete band corresponding to the viral RNA was observed. However, at the second and third passages, this band could no longer be seen, indicating that these viral particles were able to enter the cells but were not infectious and were unable to propagate.

### HHP affects viral morphology

**Figure 2:**
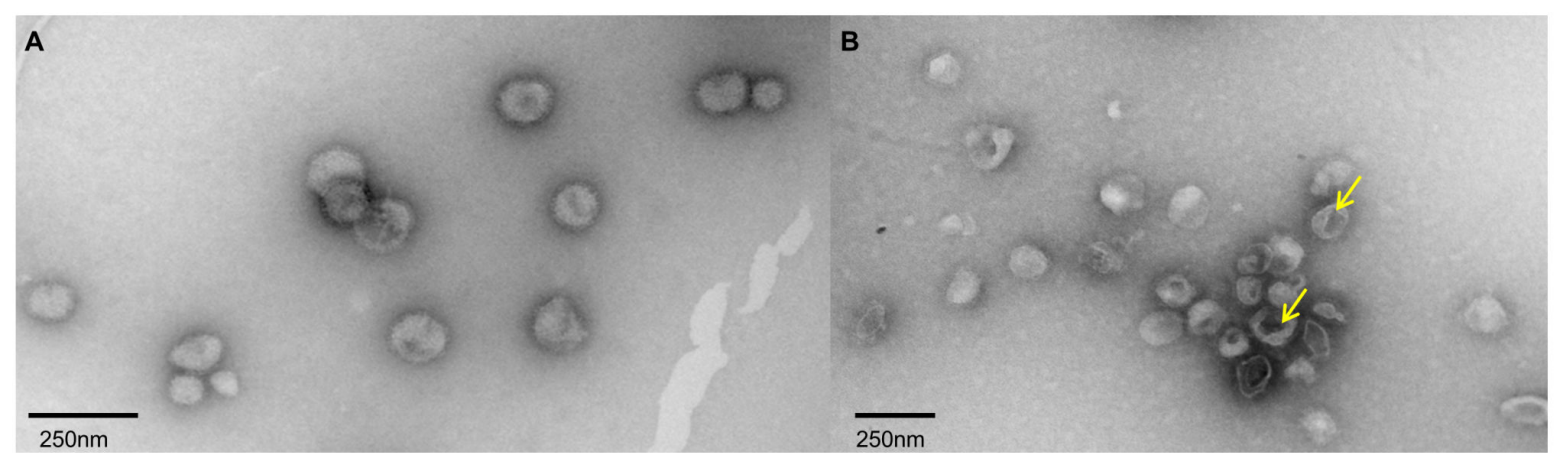
HHP affects viral morphology. (A) Control = non pressurized virus kept for 3 h at 25°C. (B) Virus pressurized for 3 h at 289.6 MPa. Arrows indicate the presence of pores.

Figure 2A and B show the transmission electron micrographs of influenza virus after incubation at atmospheric pressure or at 289.6 MPa (25°C for 3 h). The viruses treated with pressure demonstrated the same size as the native viruses but their shells were not as continuous or regular as those of the native viruses. The tails of the viral envelope (HA and NA) demonstrated no apparent modifications, and no viruses were fused. However, it is interesting to note that many of these particles seemed to contain pores in the envelope (as indicated by the arrows).

### HA is affected by pressure in a time-dependent manner but NA is not affected

**Figure 3:**
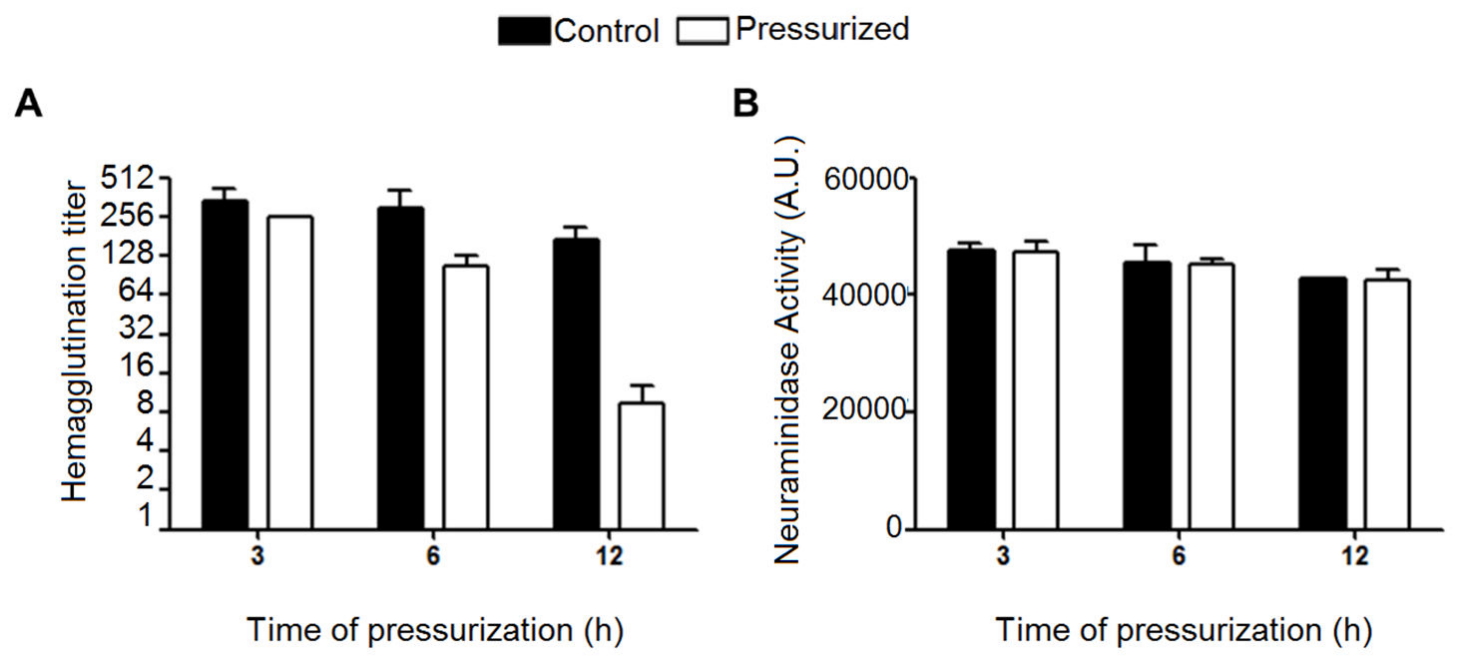
Viral glycoproteins remain functional after pressurization. (A) Hemagglutination assay titer of viruses pressurized at pH 7.4 for 3, 6, or 12 h at 289.6 MPa. Hemagglutination units were given by the reciprocal of the highest dilution where total hemagglutination was observed. (B) X-31 NA activity. Virus particles were pressurized at pH 7.4 for 3 h at 289.6 MPa. Enzymatic activity was determined with the MUNANA substrate, as described in the Materials and Methods. The NA activity was calculated by normalizing the NA activity of the pressurized virus to the NA activity of the native virus.

 To test the effects of HHP on viral binding activity, we evaluated the capacity of influenza virus to bind to erythrocytes by performing a hemagglutination assay (Figure 3A). Even after pressurization, the viruses were capable of binding to cells. A time-dependent decrease in binding activity was observed, with an apparent decrease at 3 h and a significant decrease after 12 h of pressurization.

We also investigated the other envelope glycoprotein, NA (Figure 3B). The activity of NA was evaluated by testing the cleavage of the fluorometric substrate 4-MUNANA. Interestingly, we found that this pressure range did not affect the activity of NA under the conditions tested.

### HHP preserves virus proteins structure

**Figure 4:**
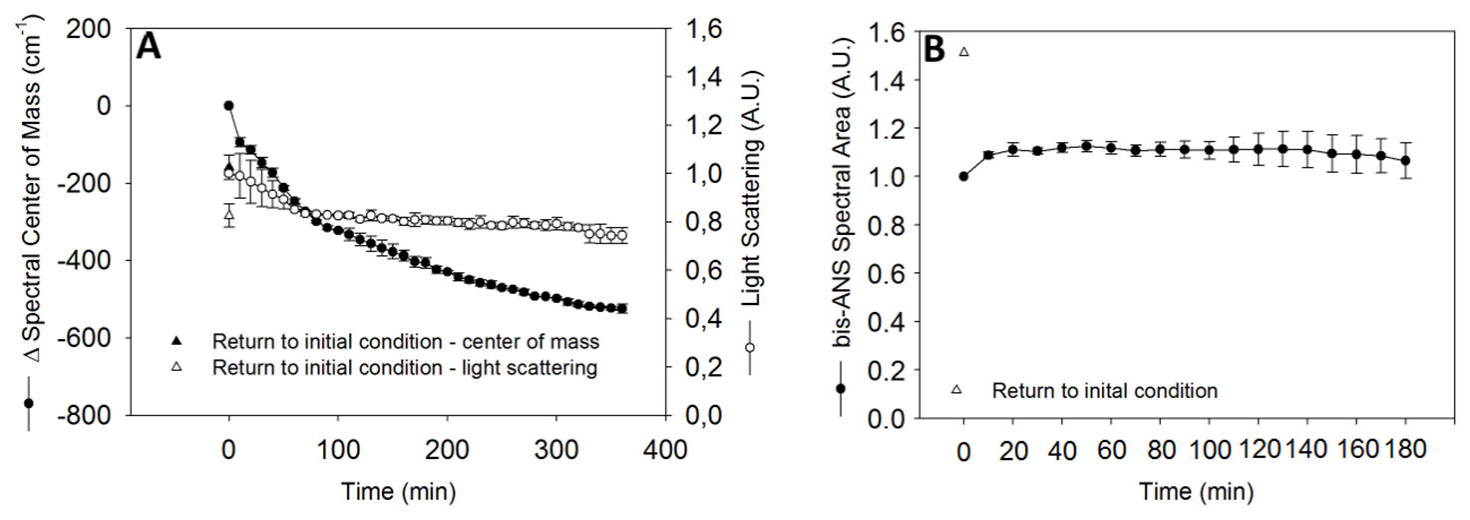
Viral proteins structure is slightly affected by HHP treatment. (A) The changes in spectral center of mass (●) and light scattering (○) were followed as a function of the pressure at 289.6 MPa over 6 h. For tryptophan fluorescence emission, the sample was excited at 280 nm, and the emission was measured at 300 to 420 nm. (B) The influenza virus was pre-incubated for 10 min with 15 mM of bis-ANS probe and then exposed to 289.6 MPa for 3 h, and the intensity of the probe was measured every 10 min.

Intrinsic and extrinsic fluorescence was used to monitor structural changes. Tryptophan residues in non-polar regions emit fluorescence when excited at 280 nm. If the protein structure is affected, tryptophan is exposed to the solvent, and this process is followed by a change in fluorescence emission that can be evaluated by the spectral center of mass. Measurements of light scattering are intended to obtain an estimate of particle size in solution. The *bis*-ANS molecule is a polarity-sensitive molecule that binds non-covalently to exposed hydrophobic segments surrounded by positively charged residues [30]. When virus was subjected to pressure, in a time-dependent manner, effects on the spectral center of mass were evident (380 cm^-1^) (Figure 4A). Moreover, the spectral center of mass showed a continuous decrease during the experiment, whereas the light scattering decreased until after 1 h of pressurization but then stabilized (Figure 4A). After returning to atmospheric pressure, the spectral center of mass returned to values that were very close to the initial values. Evaluation by bis-ANS showed that pressurization had little effect on bis-ANS fluorescence emission, indicating no time dependence for this process (Figure 4B). However, depressurization increased the fluorescence by approximately 50%, suggesting the exposure of hydrophobic regions.

Data obtained by spectroscopy showed slight changes, suggesting a good preservation of viral protein structure despite the morphological changes visualized by EM. This preservation was also suggested by the presence of functional HA and NA in pressurized viral particles. 

### Inactivated viruses partially preserve fusogenic activity

**Figure 5:**
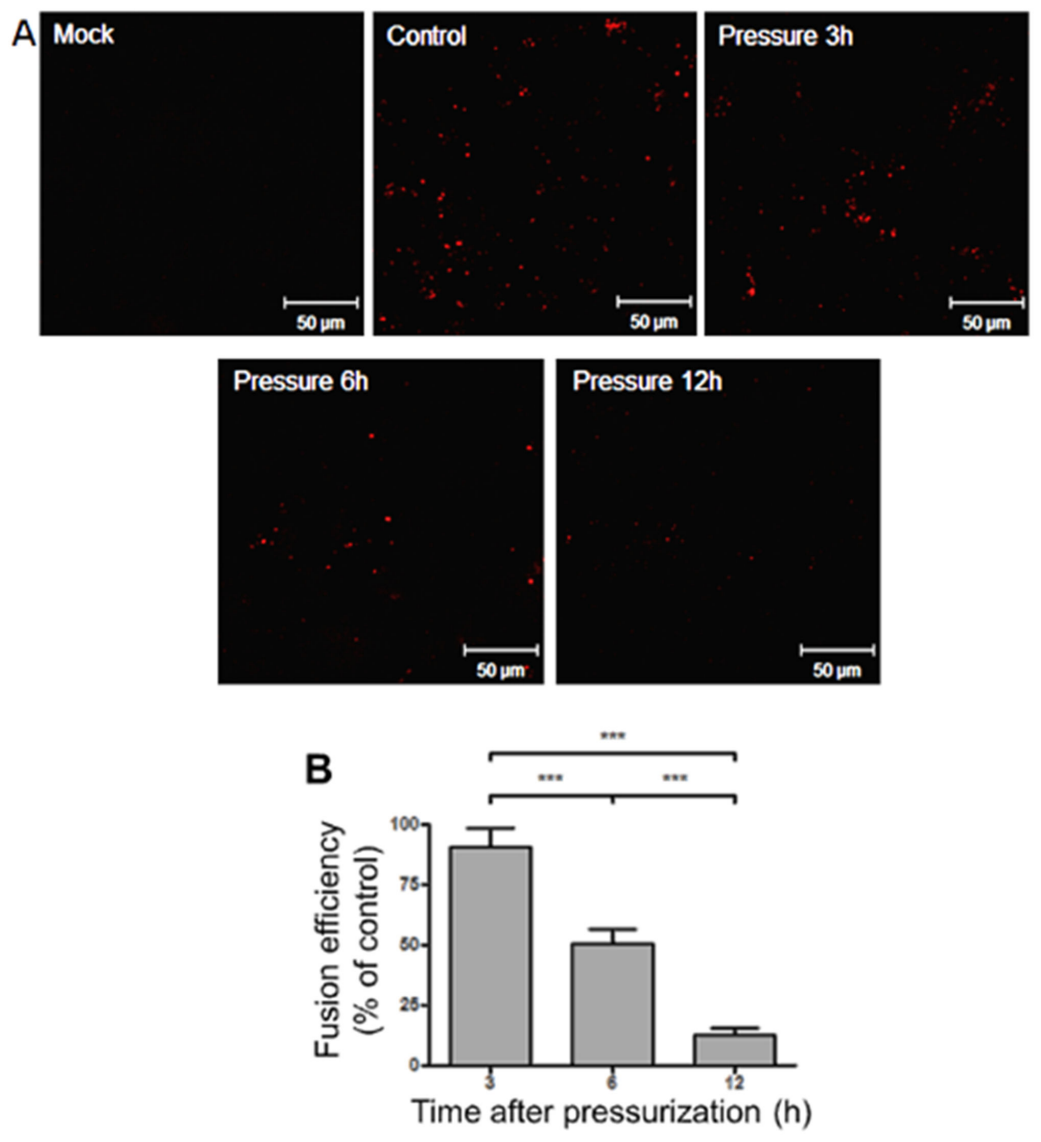
HHP treatment preserves viral fusogenic activity. Virus samples were pressurized for 3, 6, or 12 h at 289.6 MPa. (A) Viruses were incubated with DiD and monitored for their fusogenic properties. Mock (cells incubated with PBS), control (influenza viruses kept for 12 h at 25°C), and pressurized influenza virus. (B) Fusogenic activity relative to the control. The asterisks (***) mark a significant difference (***p<0.0001 by Tukey´s post-test).

After observing that pressurized viruses retained their capacity to bind to the cell surface, we investigated whether these viruses also retained the ability to fuse their membranes. The fusogenic capacity was detected at all time points (3, 6, and 12 h), and after 3 h of pressurization, the fusogenic activity was very similar to the control. Moreover, the fusogenic activity was affected by pressure in a time-dependent manner and was greatly reduced after 12 h of pressurization. The results obtained by confocal microscopy correlated with the RT-PCR results, indicating that the viruses partially maintained the capacity to fuse their membranes. This observation may explain the weak band in RT-PCR that was detected in the first passage but disappeared in subsequent passages (Figure 5). 

### Immunization with pressure-inactivated virus protects mice against infection

**Figure 6:**
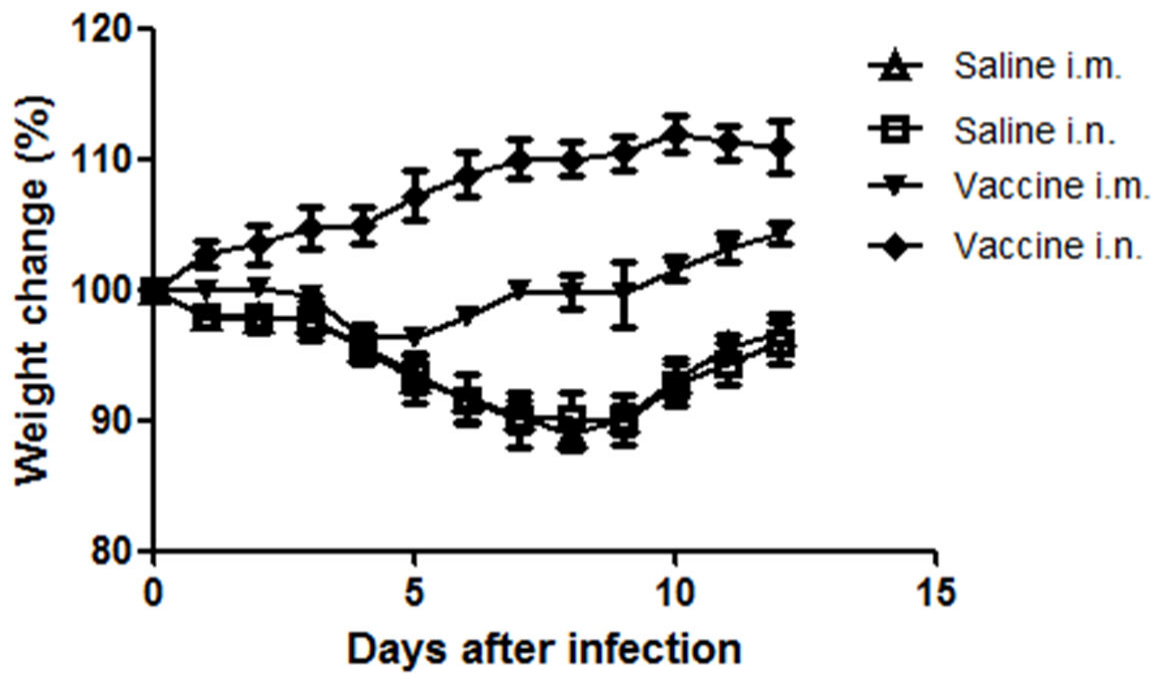
Vaccination prevents weight loss in mice. Fourteen days after the second dose, mice were i.n. challenged with 40 µl of X-31, and weight changes were observed for 12 days. Non-significant (n.s.) differences were observed between saline groups. In vaccinated groups, mice vaccinated by the i.n. route demonstrated a better response and differences were detected between both the vaccine and saline groups (p<0.0001 Tukey´s post test). Data are expressed as mean ± SD of each group of mice (n = 5 per group). i.n. – intranasal, i.m. – intramuscular.

 Mice were immunized by intramuscular (i.m.) or intranasal (i.n.) route with 40 µl (20 µg of total protein per dose). Fourteen days after the second dose, mice were challenged by the i.n. route with 40 µl of virus at a 4,096 HA titer and monitored for weight loss. In both saline groups, all mice presented weight loss (Figure 6). Weight loss and other clinical signs (data not shown) were not observed in mice immunized by i.n. route. Otherwise in mice immunized by i.m route we could observed an initial weight loss, with subsequent recovery. However, challenge of non-vaccinated animals was associated with significant weight loss (p<0.005). Weight loss monitoring was carried out with 5 animals per group.

### Vaccination with whole inactivated vaccines (WIVs) by HHP induces antibody production

**Figure 7:**
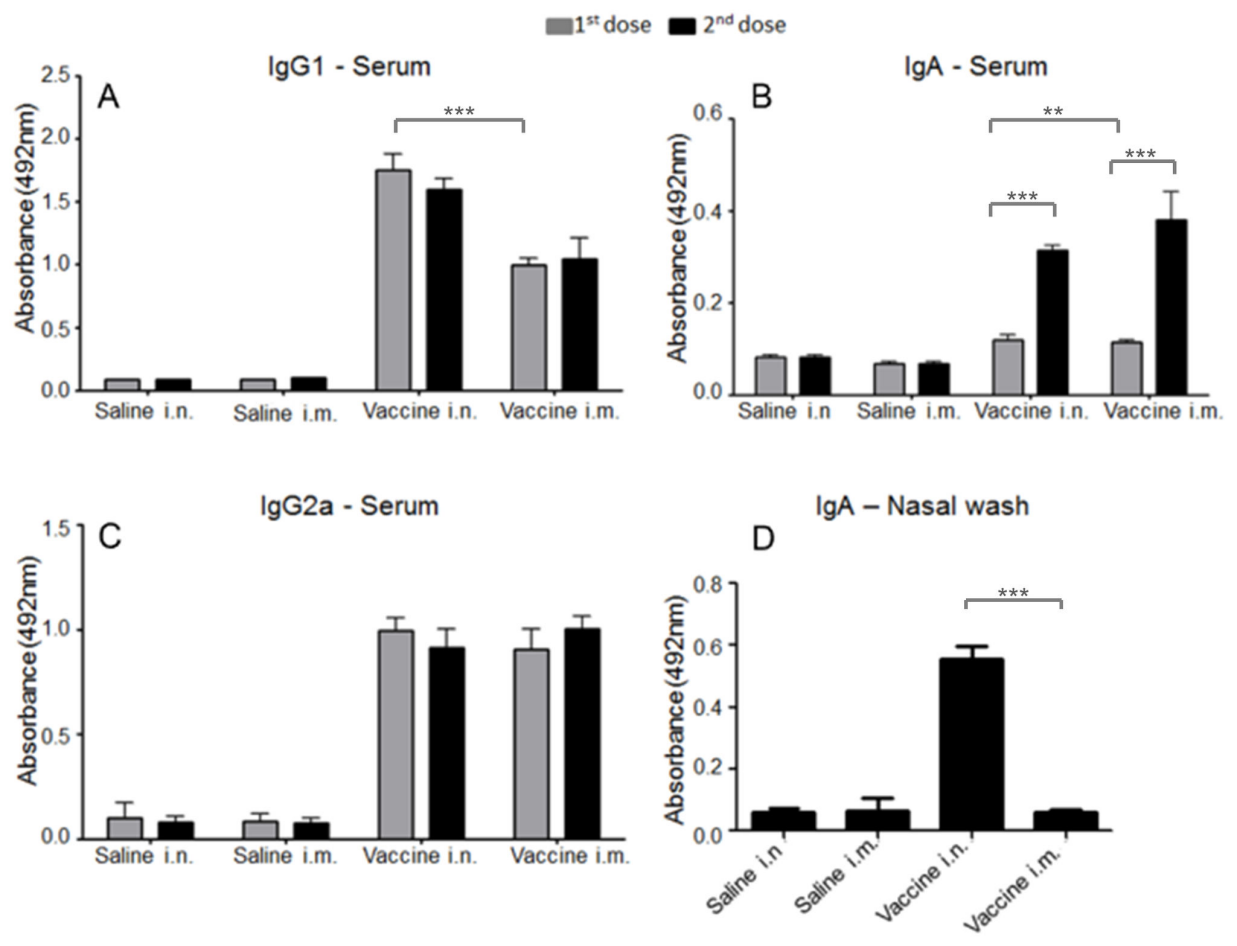
Vaccination elicits humoral response in mice. Influenza-specific IgG1, IgG2a, and IgA in the sera were measured 2 weeks after the first and second doses. (A) IgG1. (B) IgG2a. (C) IgA. (D) IgA after the second dose (nasal wash). Significant differences between the first and second doses were only observed for serum IgA. Differences in Ig levels due to route of vaccination were significant for IgG1 and serum and nasal IgA. Data are expressed as the mean ± SD of each group of mice (n = 8 per group). The asterisk (*) marks a significant difference (* p<0.05, ** p<0.01, ***p<0.0001 by one-way ANOVA with Tukey´s post-test).

 To investigate the capacity of influenza virus inactivated by pressure to induce humoral and mucosal immunity, we performed ELISA to measure serum IgG1 and IgG2a and serum and mucosal IgA antibodies specific to influenza (Figure 7). The animals received 2 doses of vaccine, but for IgG1 and IgG2a, the levels reached at the first dose were the same as for the second dose. In contrast, the serum IgA levels increased after the second dose. For IgG1, the serum antibody concentration was significantly higher in animals vaccinated by the i.n. route, and for IgG2a, the values were similar for both routes of vaccination. As expected, mucosal IgA was only elicited by the i.n. route.

### Vaccination elicits an antibody response against HA

**Figure 8:**
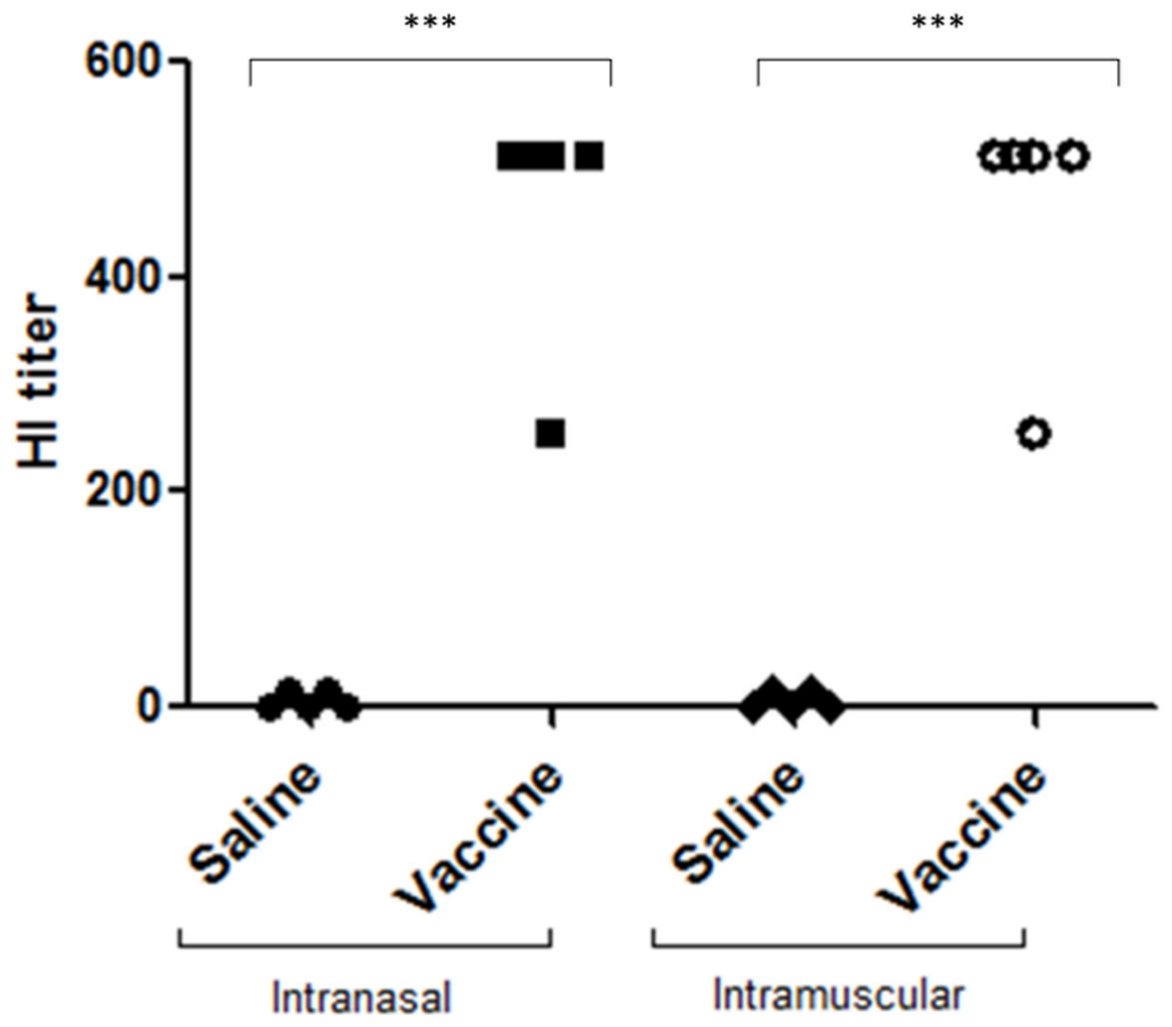
Pressurized virus induces HA antibody response. After two doses of vaccination mice presented high levels of antibodies against HA. Data are expressed from individual animal result (n = 5 per group). The serum of vaccinated mice was assessed by hemagglutination inhibition assay. The title of antibodies is referred to as the reciprocal of the highest serum dilution that resulted in complete inhibition of binding. The symbols represent the result in each animal individually. The asterisks (***) mark a significant difference (***p <0.0001 by one-way ANOVA and Tukey´s post-test).

 To investigate the capacity of vaccination to elicit an antibody response against HA, we performed HI assays. Both routes of vaccination were able to elicit an antibody response (Figure 8), which showed that vaccination induced serum antibodies able to inhibit virus binding to target cells.

## Discussion

HHP has been used as a tool to study and inactivate the structure of viruses. In this study, we evaluated the inactivation of influenza virus by HHP treatment and its effects on virus structure, activity of the viral envelope glycoproteins, fusion capacity, and potential use in vaccine formulations. We found that pressurized X-31 influenza virus completely lost its infectious capacity, with slight structural changes, maintenance of envelope glycoproteins activities, partial conservation of fusogenic activity, and protective properties as a vaccine model.

Pressure acts mainly on non-polar interactions, which determine protein folding and viral assembly. Consequently, HHP has been used as an efficient tool for virus inactivation. We observed the full inactivation of X-31 influenza viral particles following pressure treatment. Even after 3 serial passages in cell culture, no viral propagation was detected by RT-PCR. In the first passage of virus pressurized for 3 or 6 hours, a band was observed by RT-PCR (Figure 1), although this band was not observed in subsequent passages. This result corroborated our confocal microscopy findings, indicating that the virus could enter the cell but was no longer infectious. 

The effects of HHP were also evaluated by electron microscopy. Although the volume of viral particles was not altered by pressure treatment, the presence of pore formation in the viral envelope was noteworthy (Figure 2). HHP is known to decrease the lipid fluidity of biological membranes [31], which could have enabled the formation of the pores observed in the viral envelopes. This type of pressure-induced change has also been observed in other enveloped viruses, such as VSV [11]. This result shows that the evident morphological changes did not drastically affect the protein structure of the viral particles, even when they presented slight changes when analyzed by spectroscopic measurements (Figure 4). Disruptions in the viral envelope allow access to internal structures; thus, the association of these internal structures with the bis-ANS may explain the increased fluorescence of the probe, in addition to the fact that exposure of the fusion peptide also increases the fluorescence [32,33,34]. 

To analyze glycoproteins activities, we performed hemagglutination and NA assays (Figure 3A and B). HA capacity was affected in a time-dependent manner, whereas NA activity did not change. These results suggested that the structures of HA and NA were well conserved, indicating that the important epitopes involved in promoting the immunological response were preserved. The differences observed between these 2 proteins may be related to the functional sites of the glycoproteins, which are predominantly hydrophobic regions in HA and thus more sensitive to pressure, whereas in NA these regions are hydrophilic [35].

Fusion of viral membranes is a crucial step in viral infection, and we observed that fusogenic activity was affected in a time-dependent manner, ranging from 85% native efficiency in 3-h pressurized virus to 10% after 12 h (Figure 5). This shows that the majority of viruses were capable of fusing their membranes even after 3 h of pressurization. Previous studies have shown an increase in the fusogenic activity of pressurized viruses [32,33,34], although our results cannot be compared to these results because the model used to generate the previous results used liposomes, which is a simpler model when compared to the use of cells. With our model system, we demonstrated that viruses could bind and fuse to the cell membrane after pressure treatment.

The effects of hydrostatic pressure on X-31 influenza virus revealed that the structure of the particles was slightly affected by pressure treatment. However, the light scattering results showed no significant change in virus structure. Furthermore, the small variations caused by pressure treatment were partially reversible when the viral samples were depressurized, indicating that the viruses recovered most of their structure after pressure treatment (Figure 4A).

Deviations in the spectral center of mass induced by pressure treatment vary between different types of viruses, indicating different stability and susceptibility to pressure. Increasing the pressure to 289.6 MPa induced a deviation at approximately 350 cm^-1^ in human rhinovirus [36], 300 cm^-1^ in Mayaro virus [34], 150 cm^-1^ in Sindbis virus [32], 200 cm^-1^ in foot and mouth disease virus (FMDV) [37], and 690 cm^-1^ in infectious bursal diseases in chickens (IBDV) [12]. Furthermore, inactivation was reported in viruses that showed imperfect reassembly, such as Brome mosaic virus [38], and in those that reassembled when the atmospheric pressure was reestablished, such as simian rotavirus [39]. Spectroscopic investigations provide information about the structural state of viral particles, and discrete changes indicate only slight alterations and the potential for efficient immune responses due to structural epitope preservation.

When HHP was applied over time, the changes in bis-ANS remained small but increased significantly when the pressure returned to its initial state (Figure 4B). Gaspar (2002) [32] demonstrated that pressurized influenza virus exhibited an increase in bis-ANS fluorescence and suggested that this fluorescence behavior was due to fusion peptide exposure, rendering the virus in a fusogenic state, which was confirmed by performing a lipid mixing assay. The increase in bis-ANS fluorescence and the fusogenic state promoted by pressure treatment was also confirmed for other enveloped viruses such as Sindbis [32], Mayaro [34], and VSV [33]. The maintenance of fusogenic activity and exposure of the fusion peptide in WIVs by HHP is of crucial importance due to the conserved feature of this region, and this also represents a major advantage of HHP when compared to other methods of inactivation. Moreover, studies have demonstrated that antibodies against this fusion region are able to prevent infection and promote heterosubtypic protection [40,41].

Vaccination by the i.n. and i.m. routes with virus inactivated by HHP prevented disease in mice (Figure 6). Interestingly, mice vaccinated by the i.n. route demonstrated a better response than mice receiving i.m. vaccination, which is in agreement with a previous study that analyzed different routes for vaccination with influenza virus inactivated by γ radiation [42]. We believe that this result is likely due to the mucosal immunity stimulated by i.n. vaccination, which creates a barrier in the early stages of infection. This type of immunity represents a very desirable effect contributing to immune protection. Furthermore, this type of response can only be induced by vaccine models containing a conserved viral structure that is able to bind and enter cells and thus stimulate a satisfactory local immune response. Although an attenuated i.n. vaccine is currently available, this model has restrictions that hinder its application to the entire population. Thus, a low-cost inactivated vaccine based on HHP could present a safer alternative to this attenuated model.

To verify whether vaccination was able to induce the production of serum-specific Igs, we performed ELISA for influenza-specific IgG1, IgA, and IgG2a. For IgG1 and IgG2A, which are important Igs associated with viral neutralization, clearance, and survival to lethal challenge [43], no differences were observed between the first and second doses. This result suggests that a single dose of vaccine was able to induce the same level of protection as subsequent doses, and achieving such a satisfactory serological response with a single dose of a non-adjuvanted vaccine represents a desirable and promising result. A significant difference in IgG1 levels was observed between mice vaccinated by the i.n. and i.m. routes, with a better result obtained for the i.n. route. Higher levels of IgG1 have been associated with a better response to virus challenge in mice, and IgG1 is associated with immune protection of the lower respiratory tract and is the main protective mechanism of injectable vaccines [44]. However, our vaccine model demonstrated a stronger induced serum IgG1 response by the i.n. route. For IgG2a, no difference was detected between the i.n. and i.m. groups. Moreover, increased levels of IgG1 and IgG2a measured by ELISA have been more strongly correlated with vaccine efficacy than neutralization assay results alone [43].

Increased leveI of serum IgA have previously been detected in individuals demonstrating an immune response to influenza infection [45]. It has also been reported that the intravenous administration of specific IgA can be transported to intranasal secretions and protect mice against infection [46]. Mice vaccinated by the i.m. route demonstrated a stronger serum IgA response, and in this case, the second dose contributed to a further increase in the levels of IgA in both groups. As expected, mucosal IgA was elicited only by i.n. vaccination. Secretory IgA is highly desirable due its ability to eliminate a pathogen before it passes the mucosal barrier. In addition, the mucosal IgA response is particularly important for infections with highly pathogenic strains, where complications are associated with intense and uncontrolled pro-inflammatory responses [47].

HI titers are widely used to evaluate influenza virus vaccine efficacy, with a titer ≥1:40 generally used as the protective limit in humans [48]. Our results showed a protective serum level of antibodies against HA for both routes of vaccination, and this result correlated with the Ig response, indicating the production of neutralizing antibodies against the HA binding site. Thus, inactivation with the preservation of binding properties and protein structure is critical for maintaining the immunogenic epitopes of HA.

Whole influenza vaccines are superior to split and subunit vaccines and various mechanisms have been proposed to explain this difference, including a stronger Th1 response [7] and the triggering of the toll-like receptor response [8]. It is also well known that conserved internal antigens are critical for promoting heterosubtypic protection [9]. Recently, it has been demonstrated that to promote heterosubtypic protection, WIVs must preserve membrane fusion activity [49,50]. The fusogenic properties of the whole vaccine permit the release of antigens from the endosome to the cytoplasm, where they are processed and cross-presented to CD8+ T cells [49,51,52]. In a recent study, it was demonstrated that protection against lethal challenge with heterologous influenza subtypes was only efficient with a vaccine containing the whole influenza virus with preserved membrane fusion; mice vaccinated with WIVs deprived of membrane fusion preservation presented weight loss, while mice vaccinated with subunit or split vaccines died [50]. 

In addition, 2 previous studies investigated vaccination with homologous challenge (non-lethal virus) followed by heterologous challenge (lethal virus) [53,54] and showed that vaccines were efficient in protecting against homologous challenge but failed in protecting against heterologous challenge. In these same studies, non-vaccinated animals became sick when challenged with non-lethal virus but survived when challenged with lethal virus. These observations suggest that vaccination may prevent heterosubtypic immunity by protecting individuals from seasonal influenza virus. Thus, it has been suggested that in the case of an influenza pandemic, individuals who have received annual influenza vaccines would be at a higher risk to develop severe disease when compared to individuals who had been infected with seasonal influenza. However, in contrast to our model, this study used a whole inactivated virus [54] in which fusion activity was not preserved.

HHP is a well-established technique used in the food industry to eliminate bacteria from processed foods, such as canned products, milk, and juice [55]. Thus, the establishment of an HHP vaccine would not require technological innovations. Moreover, the HHP vaccine preserves fusogenic activity, which is not true of vaccines based on formalin inactivation. The exposure of the fusion peptide, as suggested by Gaspar [32], can also be an important tool to induce heterosubtypic protection. Indeed, we observed a mucosal IgA response that is generally not associated with injectable vaccines. All of these characteristics could represent great advantages for HHP vaccines. Although WIV reactogenicity commonly raises concerns, a study in humans using vaccines showed that there were no significant differences in side effects between the split vaccine and WIV groups [56]. This same study also suggested that despite the increased incidence of adverse effects in young children, the benefits of low doses may outweigh the risks. Moreover, both the WIV (Celvapan®, Baxter) and live vaccines (Flumist® and Fluenz®) are available, indicating that whole vaccines can be well tolerated. 

Although we used 2 doses of vaccine, the serum levels of IgG1 and IgG2a were not increased following the second dose, suggesting that a single dose of vaccine may be enough to confer protection. Recently an mRNA-based vaccine was shown to induce strong production of serum IgG1 and IgG2a after 2 doses of vaccination and was shown to be effective with just a single dose [57]. Similarly, our vaccine induced serum IgG1 and IgG2a responses, and this response did not change between the first and second doses, supporting the idea that a single dose was effective.

Our results indicate that HHP represents an efficient tool for inactivating entire virus particles to be used in vaccines. This type of inactivation produces an antigen with many of the chemical and physical properties of intact viral particles, which is essential for mounting a satisfactory immune response. 

Despite these promising results, further investigations are necessary to evaluate the protection level, reactogenicity, dose levels, and immunological response of an HHP-based WIV. Nonetheless, a vaccine based on pressurized virus represents a simple, fast, and low cost model that could offer an important alternative to the large-scale production of vaccines to protect against influenza, which remains a great challenge in public health.
